# Perception of change in sexual activity and sexual satisfaction in Alzheimer’s Disease: views of people with dementia and their spouse‐carers

**DOI:** 10.1002/alz.095523

**Published:** 2025-01-09

**Authors:** Marcela Moreira Lima Nogueira, Isabel Lacerda, Maria Alice Baptista, Tatiana Belfort, Marcia C. N. Dourado

**Affiliations:** ^1^ Federal University of Rio de Janeiro, Rio de Janeiro, Rio de Janeiro Brazil; ^2^ Federal University of Rio de Janeiro, Rio de Janeiro Brazil

## Abstract

**Background:**

The progression of dementia may be followed by decreased sexual activity for People with Alzheimer’s Disease (PwAD) and their spouse‐carers. We designed this study to investigate the perception of change in sexual activity and sexual satisfaction among couples whose spouses were diagnosed with Alzheimer’s Disease (AD).

**Method:**

Using a cross‐sectional design, we compared 74 dyads of people with Alzheimer´s disease (PwAD) and their spouse‐carers, and 21 elderly dyads control. We assessed sexual satisfaction with Questionnaire on Sexual Experience and Satisfaction (QSES), cognition using a Mini‐Mental State Examination (MMSE), disease severity using a Clinical Dementia Rating scale (CDR), awareness of disease with Assessment Scale of Psychosocial Impact of the Diagnosis of Dementia (ASPIDD), functionality with Pfeffer Functional Activities Questionnaire (FAQ), depressive symptoms with Cornell Scale for Depression in Dementia (CSDD), quality of life using a Quality of Life in Alzheimer’s Disease Scale (QoL‐AD), and burden using a Zarit Burden Interview (ZBI). Univariate and multivariate regression analyses were conducted to identify the factors that influenced couples’ sexual satisfaction.

**Result:**

We found a significant difference between the perception and no perception of change in sexual activity of PwAD (*p <* 0.001), spouse‐carers (*p <* 0.01), and controls (*p <* 0.05). Moderate to severe sexual dissatisfaction was observed in 36.5% of PwAD, 65% of spouse‐carers, and 31% of controls. PwAD sexual satisfaction was related to cognitive impairment (*p <* 0.05). Spouse‐caregivers sexual satisfaction was related to gender (*p <* 0.05) and the presence of sexual activity (*p <* 0.001).

**Conclusion:**

The perception of change and consequent interruption of sexual activity, with higher sexual dissatisfaction, were higher in PwAD and their spouse‐carers, in comparison with control group. We also found that spouse‐carers experienced lower levels of sexual satisfaction than PwAD and healthy elderly couples. Moreover, PwAD sexual satisfaction was related to the level of cognitive impairment and spouse‐carers’ sexual satisfaction was related to gender and the presence of sexual activity.

